# Examining the Effectiveness of Gamification in Mental Health Apps for Depression: Systematic Review and Meta-analysis

**DOI:** 10.2196/32199

**Published:** 2021-11-29

**Authors:** Stephanie G Six, Kaileigh A Byrne, Thomas P Tibbett, Irene Pericot-Valverde

**Affiliations:** 1 Department of Psychology Clemson University Clemson, SC United States; 2 SAP National Security Services, Inc Newtown Square, PA United States; 3 Clemson University School of Health Research Greenville, SC United States

**Keywords:** depression, reward, gamification, mental health apps, apps

## Abstract

**Background:**

Previous research showed that computerized cognitive behavioral therapy can effectively reduce depressive symptoms. Some mental health apps incorporate gamification into their app design, yet it is unclear whether features differ in their effectiveness to reduce depressive symptoms over and above mental health apps without gamification.

**Objective:**

The aim of this study was to determine whether mental health apps with gamification elements differ in their effectiveness to reduce depressive symptoms when compared to those that lack these elements.

**Methods:**

A meta-analysis of studies that examined the effect of app-based therapy, including cognitive behavioral therapy, acceptance and commitment therapy, and mindfulness, on depressive symptoms was performed. A total of 5597 articles were identified via five databases. After screening, 38 studies (n=8110 participants) remained for data extraction. From these studies, 50 total comparisons between postintervention mental health app intervention groups and control groups were included in the meta-analysis.

**Results:**

A random effects model was performed to examine the effect of mental health apps on depressive symptoms compared to controls. The number of gamification elements within the apps was included as a moderator. Results indicated a small to moderate effect size across all mental health apps in which the mental health app intervention effectively reduced depressive symptoms compared to controls (Hedges g=–0.27, 95% CI –0.36 to –0.17; *P*<.001). The gamification moderator was not a significant predictor of depressive symptoms (β=–0.03, SE=0.03; *P*=.38), demonstrating no significant difference in effectiveness between mental health apps with and without gamification features. A separate meta-regression also did not show an effect of gamification elements on intervention adherence (β=–1.93, SE=2.28; *P*=.40).

**Conclusions:**

The results show that both mental health apps with and without gamification elements were effective in reducing depressive symptoms. There was no significant difference in the effectiveness of mental health apps with gamification elements on depressive symptoms or adherence. This research has important clinical implications for understanding how gamification elements influence the effectiveness of mental health apps on depressive symptoms.

## Introduction

Depression is a highly prevalent mental disorder in the United States that affects 17.3 million people [1]. Effective treatments are available to treat depression, including pharmacotherapy and psychological treatment [2,3]. However, widespread barriers to treatment exist, such as problems of consistent adherence, access to mental health care resources, and cost [4-7]. The United States alone spends approximately US $71 billion annually on depression treatment [8], which underscores the substantial financial burden that depression can incur. Furthermore, of all adult Americans who experienced a major depressive episode in 2017, 35% did not receive treatment [1].

To mitigate these challenges, companies have created technology-based mental health apps, with the goal of alleviating symptoms of various mental disorders [8,9]. As of 2019, 81% of all Americans owned a smartphone, which highlights the potential impact of technology-based mental health apps: providing a platform, consuming less time, requiring less commitment, and allowing users to move at their own pace [10]. This ubiquity suggests that app technology could make treatment more accessible by providing individuals with cost-efficient tools and apps to aid them between sessions. However, development of these apps is recent and additional attention is needed to identify what is most effective and rewarding about these digital tool kits.

People with depression frequently experience anhedonia, which may result in blunted sensitivity to reward [11-15]. Depressed individuals view rewards, like money or social encouragement, as less motivating than individuals without depressive symptoms [16-18]. Effective therapeutic approaches may benefit from improving this reward-processing deficit, potentially through the use of gamification elements. Gamification is defined as the use of game-design elements and incentives combined with desired behaviors in order to positively influence user motivation, behavior of users, and adherence [19-22]. Previous research suggests that different gamification elements represent motivational affordances that can influence psychological outcomes [23]. These elements include leaderboards, achievements, badges, levels, challenges, and points [23]. The Unified Gamification and Motivation (UGM) model lends a framework for understanding how including gamification elements in therapeutic intervention can enhance treatment engagement [24]. Based on this model, the inclusion of game-like elements would make the intervention more salient, which could increase motivation to use the intervention, thereby resulting in greater treatment usage.

Much of the work demonstrating the effects of gamification elements on reward motivation stems from video game research. For example, in a recent study, young adults were randomized to either a video game training or a control task [25]. After 2 months playing the video game, participants randomized to the video game intervention exhibited higher activation in the ventral striatum (ie, increase in reward activation) during a nongamified task than those assigned to the control task at 2 months posttest. This finding suggests that (1) the effect of video games on reward motivation can transfer to other tasks and (2) video games can enhance individuals’ general reward responsiveness to positive stimuli [25]. However, very little research has centered on how individuals suffering from depression are motivated to pursue reward and engage in video games. The capability of video games to enhance reward processing, motivation, and engagement could play a critical role in the development of app technology specifically grappling with anhedonia.

Mental health apps provide a potential way to reduce symptoms and increase adherence by dispensing psychoeducation and other therapeutic skills through an electronic, easily accessible format [26-34]. Some apps include reward-based features, in the form of money, games, or hearts (eg, SPARX-R) [35], while other apps do not mention any type of reward (eg, AI Tess) [36]. Yet, these apps appear effective overall; in an initial meta-analysis collecting data from nine randomized controlled trials (RCTs) with depression as a secondary concern (mean age 36.1 years; male, 34.8%), results indicated that mental health apps led to a large reduction in depressive symptoms [30]. A second meta-analysis (19 RCTs; mean age 30.7 years; female, 63.17%) examined the effect of smartphone mental health apps on a variety of disorders (eg, anxiety, substance use, and sleep problems) including depression, and results showed significant differences between groups in reducing depressive symptoms with a small to moderate effect size [37]. Another meta-analysis, which predominately recruited adults over the age of 16 years (93.3%), examined 45 RCTs with various technological interventions for depressive patients, like symptom tracking, online diaries, and email and phone reminders. Depressive symptoms showed significant reductions in comparison to either wait-list or treatment-as-usual controls [38]. Taken together, there is evidence that mental health apps are effective, but the variability in effect size between meta-analyses suggest there could be another mechanism. Given the UGM model, gamification is a logical next step to replicate prior meta-analyses and add further context. Mental health apps offer a novel, easily accessible way to combine therapy techniques and motivational reward elements, like a video game, yet it is unclear whether reward or gamification features uniquely offer additional advantages in reducing depressive symptoms. Pairing this novel app approach with traditional techniques that are effective for depression may be able to mitigate anhedonia symptoms.

The purpose of this systematic review and meta-analysis is to (1) provide a comprehensive and updated meta-analytical evaluation of the effectiveness of mental health apps in reducing depressive symptoms and (2) to assess whether mental health apps with gamification elements are more effective than those without. Prior research on video games, like apps, indicates high levels of reward motivation and pleasure [25,39]. Additionally, previous systematic reviews and meta-analyses have shown significant reductions in symptoms for depression and other mental disorders while using mental health apps, some of which included cognitive behavioral therapy (CBT) or gamification elements [26,36,37,40,41]. However, no study to date has explored the effectiveness of mental health apps with gamification components in mitigating depressive symptoms.

Previous research shows that gamification can increase motivation to engage with mental health apps [42-44], improve mood [45], and activate the ventral striatum, which can enhance individuals’ general reward responsiveness to positive stimuli [21]. Building on this research, we propose that gamification may enhance the efficacy of therapeutic-based apps (eg, CBT) and reduce depressive symptoms through the following mechanism: (1) it might increase engagement with and adherence to mental health apps and (2) it may activate reward-mediated neural pathways, eliciting positive feelings, which might counteract some negative feelings from depression. We hypothesize that mental health apps that include gamification elements will be more effective in reducing depressive symptoms and increasing adherence than those without such elements.

## Methods

### Overview

The systematic review and meta-analysis were conducted following the PRISMA (Preferred Reporting Items for Systematic Reviews and Meta-Analyses) guidelines [46]. A protocol was designed and registered through the Open Science Framework after data collection and before data extraction and analysis began [47]. The quality of the studies included was assessed through the Cochrane Collaboration’s risk of bias assessment tool [48].

### Eligibility and Inclusion Criteria

Studies were included if they met the following criteria: (1) studies involving human participants; (2) mental health apps targeting depression as a primary, secondary, or tertiary outcome; (3) RCTs or experimental or quasi-experimental designs with an active, wait-list, or treatment-as-usual control group; (4) published between January 1, 2005, and December 31, 2020; (5) mental health app intervention groups contain elements of CBT, acceptance and commitment therapy (ACT), behavioral activation (BA), or mindfulness; and (6) provide a measure of depressive symptoms pre- and posttreatment. Studies were excluded if they (1) were books chapters, meta-analyses, reviews, case studies, or opinion pieces; (2) were not written in English; (3) included participants younger than 18 years of age; (4) included participants with a terminal or life-threatening illness (eg, patients with cancer who had depression) to avoid potential confounds of disentangling which condition (ie, life-threatening illness or depression) influenced outcomes; and (5) included a therapist or other mental health specialist’s guidance for the mental health apps, as this could create a confound. No specific measure of depression was required, in order to allow the focus to remain on the mental health apps.

### Literature Search Strategy

Studies were identified through a comprehensive literature search in PubMed, PsycInfo, Cochrane Clinical Trials Registry, Web of Science, and PsyArXiv (for publication bias) with no publication date restriction. The search was conducted in February 2021. Additionally, the authors conducted a manual search to locate studies that were not identified through databases. Search terms used three different concepts critical to the extant literature: app-based, mental health, and reward or gamification. Within each concept (eg, app-based), we identified multiple tags that reflected this concept (eg, “mental health app” and “MHapp”). The specific combination of operators can be found in Multimedia Appendix 1. This resulted in 129 unique combinations of search terms (eg, “MHapp-Money” and “Depression-Points”).

### Study Selection Procedure

During the identification phase, articles were identified and collected based on the search term combinations from the five databases. After duplicate removal, two researchers (SGS and KAB) independently conducted initial screening for eligible articles by assessing titles and abstracts for inclusion or exclusion criteria (Multimedia Appendix 2). After the initial screening, both researchers independently assessed the remaining full-text articles against the inclusion and exclusion criteria. Disagreements were resolved through re-examination of the articles in question and discussion among the screeners.

### Data Extraction

Two independent reviewers independently coded the studies in a Microsoft Excel spreadsheet. The following data were extracted from each article: first author, year of publication, participants’ characteristics (ie, gender and age), population, and study length (Multimedia Appendix 3), as well as app name, app classification (ie, mobile or internet), presence of gamification elements, type of gamification element (eg, digital rewards, challenge or game, and competition or challenges), app adherence, the instrument used to measure depression, and type of therapy (eg, CBT and ACT) offered (Table 1) [31,49-85].

**Table 1:** Study app classification, therapy, and gamification information.

First author, publication year, and app		App classification	Depression measure	Therapy intervention	Game elements, n	Adherence rate, %
**Bakker, 2018 [54]**						
	MoodMission	Mobile	PHQ-9^a^	CBT^b^	2	69.6
	MoodPrism	Mobile	PHQ-9	CBT	2	46.4
	MoodKit	Mobile	PHQ-9	CBT	0	46.0
**Berger, 2011 [85]**						
	Deprexis	Mobile	BDI-II^c^	BA^d^, PST^e^, and mindfulness	1	N/A^f^
**Birney, 2016 [80]**						
	Moodhacker	Internet	PHQ-9	Mindfulness	0	N/A
**Bosso, 2020 [67]**						
	Headspace	Mobile	DASS-21^g^	Mindfulness	3	58.0
**Bostock, 2019 [68]**						
	Headspace	Mobile	HADS^h^	Mindfulness	3	2.0
**Botella, 2016 [57]**						
	Smiling is Fun	Internet	BDI-II	CBT	1	86.4
**Choi, 2012 [81]**						
	Brighten Your Mood	Internet	CBDI^i^	CBT	0	68.0
**Collins, 2018 [51]**						
	MindWise	Internet	PHQ-9	CBT	0	41.7
**Dahne, 2019 [66]**						
	Aptivate	Mobile	BDI-II	CBT	2	36.4
	iCouch CBT	Mobile	BDI-II	CBT	0	N/A
**Dahne, 2019 [77]**						
	Moodivate	Mobile	BDI-II	CBT	2	42.9
	Moodkit	Mobile	BDI-II	BA	0	N/A
**Deady, 2020 [78]**						
	HeadGear	Internet	PHQ-9	BA and mindfulness	2	10.1
**de Graaf, 2009 [62]**						
	Colour Your Life	Internet	BDI-II	CBT	0	36.0
**Fish, 2019 [53]**						
	Headspace	Mobile	PHQ-9	Mindfulness	3	N/A
**Flett, 2018 [69]**						
	Headspace	Mobile	CES-D^j^	Mindfulness	3	16.4
	Smiling Mind	Mobile	CES-D	Mindfulness	1	15.4
	Evernote	Mobile	CES-D	N/A	0	N/A
**Fuller-Tyszkiewicz, 2020 [73]**						
	StressLess	Mobile	DASS-21	Mindfulness-based CBT	1	19.0
	Stress Monitor	Mobile	DASS-21	N/A	1	N/A
**Gilbody, 2015 [63]**						
	Beating the Blues	Internet	PHQ-9	CBT	0	79.0
	MoodGYM	Internet	PHQ-9	CBT	2	75.0
**Ha, 2020 [49]**						
	Spring	Mobile	BDI-II	CBT	1	N/A
**Howells, 2016 [56]**						
	Headspace	Mobile	CES-D	Mindfulness	3	29.8
	Catch Notes	Mobile	CES-D	N/A	0	N/A
**Hur, 2018 [72]**						
	Todac Todac	Mobile	BDI-II	CBT	3	N/A
**Kladnitski, 2020 [71]**						
	iCBT^k^ program	Mobile	PHQ-9	CBT and mindfulness	2	69.4
	MEiCBT^l^ program	Mobile	PHQ-9	CBT and mindfulness	2	69.7
	iMT^m^ program	Mobile	PHQ-9	CBT and mindfulness	2	67.6
**Krafft, 2019 [58]**						
	Simple Matrix	Internet	DASS-21	ACT^n^	1	42.9
	Complex Matrix	Internet	DASS-21	ACT	2	40.0
**Levin, 2020 [75]**						
	Stop, Breathe, & Think	Internet	CCAPS-34^o^	Mindfulness	0	63.0
**Lintvedt, 2013 [79]**						
	MoodGYM	Mobile	CES-D	CBT	2	N/A
	Blue Pages	Mobile	CES-D	N/A	0	N/A
**Löbner, 2018 [64]**						
	MoodGYM	Internet	PHQ-9	CBT	2	13.0
**Lokman, 2017 [60]**						
	CDMIs^p^: Sleep Better, Worry Less, and Stress Less	Mobile	IDS-SR^q^	CBT	1	N/A
**Lüdtke, 2018 [59]**						
	Be Good to Yourself	Internet	PHQ-9	CBT	3	79.6
**Mantani, 2017 [52]**						
	Kokoro	Mobile	BDI-II	CBT	6	40.7
**McCloud, 2020 [70]**						
	Feel Stress Free	Internet	HADS	CBT and mindfulness	2	7.0
**Moberg, 2019 [61]**						
	Pacifica	Internet	DASS-21	CBT and mindfulness	3	N/A
**Montero-Marín, 2016 [82]**						
	Smiling is Fun	Internet	BDI-II	CBT	1	84.3
**Richards, 2020 [55]**						
	Space from Depression	Internet	PHQ-9	CBT	2	N/A
**Richards, 2015 [83]**						
	Space from Depression	Internet	BDI-II	CBT	2	36.0
**Roepke, 2015 [31]**						
	SuperBetter	Internet	CES-D	CBT	7	45.6
**Rollman, 2018 [84]**						
	Beating the Blues	Internet	PROMIS^r^	CBT	0	85.8
**Schure, 2019 [76]**						
	Thrive	Mobile	PHQ-9	CBT	3	58.6
**Sethi, 2013 [65]**						
	MoodGYM	Mobile	DASS-21	CBT	2	N/A
**Tighe, 2017 [74]**						
	ibobbly	Mobile	PHQ-9	Mindfulness and ACT	0	85.0
**Twomey, 2014 [50]**						
	MoodGYM	Internet	DASS-21	CBT	2	27.3

^a^PHQ-9: 9-item Patient Health Questionnaire.

^b^CBT: cognitive behavioral therapy.

^c^BDI-II: Beck Depression Inventory-II.

^d^BA: behavioral activation.

^e^PST: problem-solving therapy.

^f^N/A: not applicable; values were not reported.

^g^DASS-21: 21-item Depression, Anxiety, and Stress Scale.

^h^HADS: Hospital Anxiety and Depression Scale.

^i^CBDI: Chinese version of the Beck Depression Inventory.

^j^CES-D: Center for Epidemiological Studies Depression Scale.

^k^iCBT: internet-delivered cognitive behavioral therapy.

^l^MEiCBT: mindfulness-enhanced internet-delivered cognitive behavioral therapy.

^m^iMT: internet-delivered mindfulness training.

^n^ACT: acceptance and commitment therapy.

^o^CCAPS-34: Counseling Center Assessment of Psychological Symptoms-34.

^p^CDMI: complaint-directed mini-intervention.

^q^IDS-SR: Inventory of Depressive Symptomatology Self-Report.

^r^PROMIS: Patient-Reported Outcomes Measurement Information System.

The conceptualization of gamification was modeled after previous research in which gamification was defined as having three components: a design feature that uses motivational affordances to influence psychological and behavioral outcomes [23]. Another literature review that focused exclusively on the health and well-being app domain has described very similar conceptualizations of gamification elements [86]. Modeled after these gamification literature reviews, gamification for this meta-analysis was defined using the following nine motivational affordance categories: points, achievements or badges, levels, narrative stories or themes, clear goals, performance-based feedback, rewards, progress metrics (eg, progress bars), and challenges [23,86]. While gamification can also include leaderboards [19], this element was excluded from the meta-analysis, as leaderboards in the context of mental health may promote social comparison, which can be counterproductive [87,88]. First authors of the studies were contacted via email to confirm conceptualization of the number of gamification elements. The number of gamification elements included in each intervention was included as a moderator in analyses.

Raw depression scores (mean and SD) at posttreatment for each study were extracted. If a study compared more than one mental health app intervention to a control group, or if more than one independent sample was examined in an article, both were included as separate comparisons. Studies used different, but convergently valid, measures of depression. If the articles met any of the exclusion criteria, specifically missing data, they were excluded from data analysis (n=12).

### Quality Assessment

**Figure 1:**
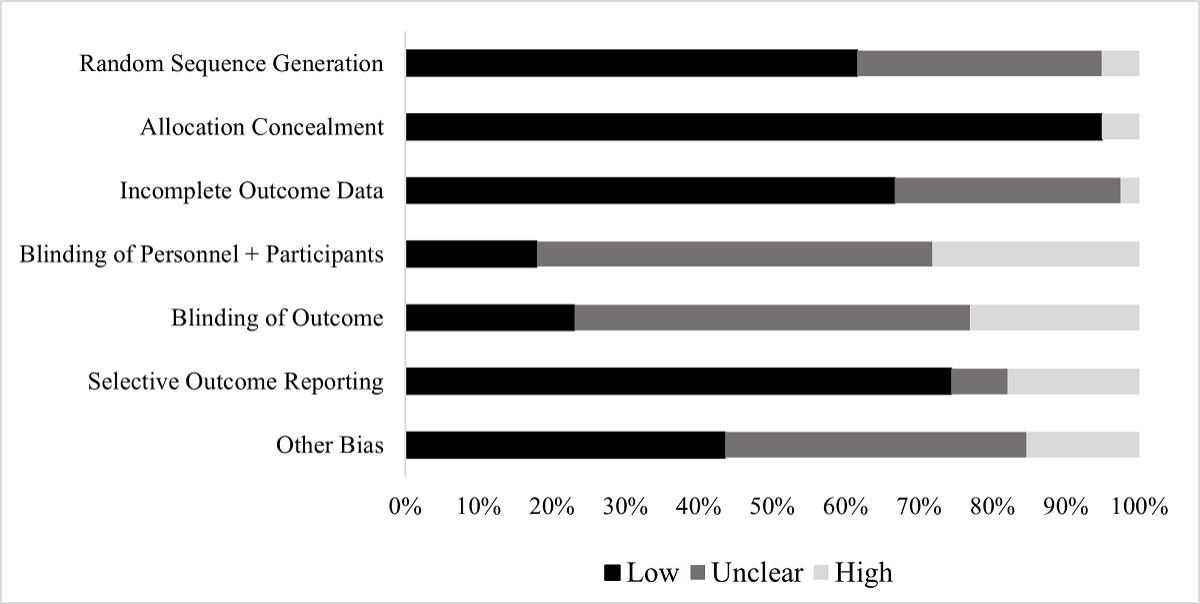
Risk of bias across all studies included in the meta-analysis based on the Cochrane Collaboration's risk of bias assessment tool.

The quality of each study was assessed using the Cochrane Collaboration’s risk of bias assessment tool, which provided seven basic criteria: random sequence generation, allocation concealment, blinding of participants and personnel, blinding of outcome assessment, incomplete outcome data, selective reporting, and other biases [48]. Studies were scored on a scale ranging from 0 to 2, where 0 indicates “low or no bias,” 1 indicates that the “level of bias is unclear,” and 2 indicates “high bias” (Multimedia Appendix 4). In line with previous research, if a study did not address one of the categories, it was given a 1 for the lack of explanation [89]. Total scores for individual and all studies are presented in Figure 1.

### Data Analysis

#### Overview

The resulting depression questionnaires included in the analyzed studies were as follows: the 9-item Patient Health Questionnaire (PHQ-9) [90]; the Beck Depression Inventory-II (BDI-II) [91]; the Center for Epidemiological Studies Depression Scale (CES-D) [92]; the depression subscale of the 21-item Depression, Anxiety, and Stress Scale (DASS-21) [93]; the Hospital Anxiety and Depression Scale (HADS) [94]; the Inventory of Depressive Symptomatology Self-Report (IDS-SR) [95]; the depression scale from the Counseling Center Assessment of Psychological Symptoms-34 (CCAPS-34) [96]; and the depression scale from the Patient-Reported Outcomes Measurement Information System (PROMIS). All questionnaires involved a 4-point scale, ranging from 0 (very low levels of depressive symptoms) to 3 (very high levels of depressive symptoms). Some studies reported sum scores, while others reported average scores. To ensure that all questionnaire data were comparable along the same scale, average depression scores were computed for analysis.

The meta-analytic data were analyzed in RStudio, primarily using the meta (version 4.18) and metafor (version 3.0) packages in R (version 3.6.3; The R Foundation) to determine effect size and between-group differences. Means and variances were aggregated for studies that included two primary measures of depression to compute a single comparison [97]. This led to a total of 50 comparisons in the meta-analysis. From this data, the pooled SD, *t* test value, P value, degrees of freedom, SE, and Hedges g were calculated. The Hedges g effect size provides an index of the magnitude of the difference between two means and corrects for potential biases in small samples [97,98]. The bias correction was performed using the following formula: 
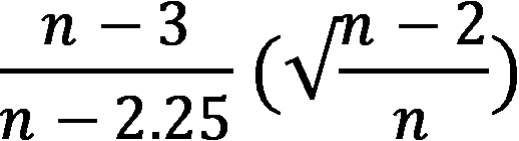
 where n = n_1_ + n_2_ [99]. An effect size of 0.2 represents a small effect size, 0.5 reflects a moderate effect size, and ≥0.80 represents a large effect size [98,100].

Following previous meta-analyses comparing intervention effects on depressive symptoms [26,37,41], the effectiveness of mental health apps was assessed using one outcome: difference in depressive symptoms between intervention and control groups at posttreatment. To test the hypothesis that mental health apps would be effective in reducing depressive symptoms, a random effects model was used to examine differences in the magnitude of depressive symptomology between those mental health app interventions compared to control conditions. The continuous variable of number of gamification elements was included in the random effects model as a moderator to test whether gamification elements influenced the effectiveness of mental health apps. The duration of the intervention, in months, was also included as a moderator variable. The I^2^ statistic was computed to determine heterogeneity across studies: an I^2^ value of ≤25% suggests low heterogeneity, ~50% suggests moderate heterogeneity, and ≥75% suggests high heterogeneity across studies [101]. While some articles provided follow-up time point data, only data from the postintervention period or data that were specified as the primary endpoint were analyzed in the primary meta-analysis.

#### Subgroup and Sensitivity Analyses

A sensitivity analysis using a random effects model with the gamification moderator was conducted to examine the effectiveness of mental health apps on depressive symptoms among the CBT-based apps that excluded ACT and mindfulness-based interventions. A secondary meta-regression analysis with the gamification moderator was performed for studies that included a measure of adherence rates (ie, percentage completion of all intervention modules or requirements) for the intervention condition. The adherence analysis included 28 studies with 37 comparisons.

#### Assessment of Publication Bias

A funnel plot was created to provide a visual of potential bias. The vertical line indicates the estimated effect of all studies. Pseudo-CIs were generated around this line in homogenous data sets to indicate 95% CI boundaries. Asymmetrical funnel plots suggest that the effects of an intervention in studies with small sample sizes are different—typically more impactful—than in studies with larger sample sizes and may indicate publication bias [102]. However, if model estimates suggest heterogeneity, a transformation manipulates these pseudo-CIs to take the heterogeneity into account: ±1.96 × √(SE^2^ + τ^2^) where the τ^2^ variable indicates the degree of heterogeneity. Its inclusion in the pseudo-CI calculation results in two curved lines asymptotic to the original estimated effect, a broader and wider funnel more inclusive of variance.

The presence of publication bias was also measured with the Egger test and the trim-and-fill approach by Duval and Tweedie. The Egger test [103] was performed to quantify whether there was significant small-study publication bias in the included studies. The trim-and-fill analysis by Duval and Tweedie was conducted to establish an unbiased estimate of the pooled effect size by correcting for funnel plot asymmetry due to publication bias [104]. Significant findings indicate whether the study sample is asymmetrical or “missing” publications that would positively or negatively bias the estimate.

## Results

### Results of the Review

**Figure 2:**
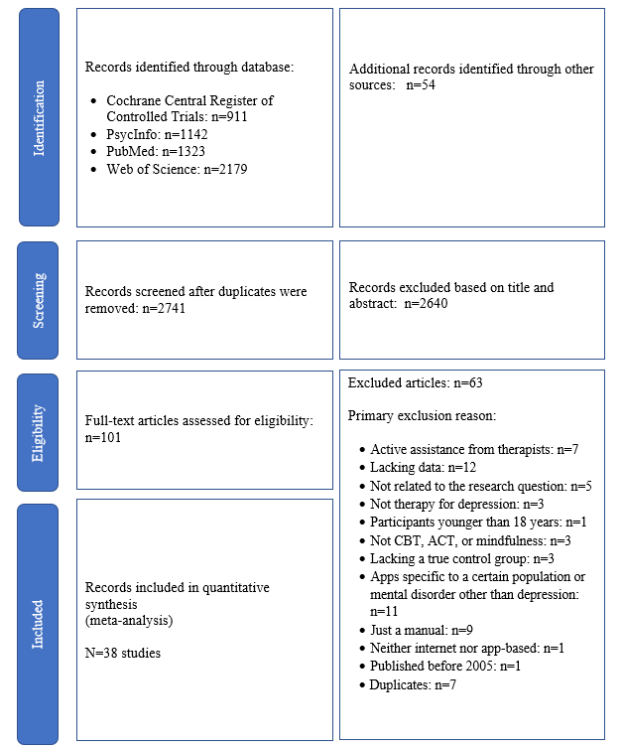
PRISMA (Preferred Reporting Items for Systematic Reviews and Meta-Analyses) flow diagram of the studies included in the systematic review and meta-analysis. ACT: acceptance and commitment therapy; CBT: cognitive behavioral therapy.

Data collection commenced in February 2021 and ended in May 2021. As of June 2021, a total of 5597 articles were identified through the literature search. After duplicate removal, 2741 eligible articles remained for title and abstract screening. Two researchers (SGS and KAB) independently identified 101 articles as potentially eligible. Screeners had an agreement rate of 98.94% (Cohen *k*=0.85). Full-text screening of these 101 articles was conducted. Of the reviewed articles, 38 studies with 39 different mental health apps met the inclusion criteria and were therefore included. Figure 2 presents the study selection identification, screening, and eligibility process.

### Participant and Study Characteristics

A total of 8110 participants provided analyzable data (4362, 53.8%, in the interventions and 3748, 46.2%, in the control conditions) for this meta-analysis, with the majority of participants being female (n=4728, 58.3%; mean age 35.6, SD 7.9, years). Multimedia Appendix 3 shows descriptive information for each study. Treatment duration ranged from 10 days to 4 months. Most of the studies (26/38, 68%) used mental health apps with CBT [31,45-48,50,51,53,55-62,66-68,72-75,77-79], 32% (12/38) used mindfulness [49,52,57,63-67,69-71,74,76,82], 8% (3/38) used ACT [62,73,82], 8% (3/38) did not have a therapy associated with the app, and only 5% (2/38) used BA [54,70]. In terms of the depressive symptom outcome measures, 32% (12/38) of the studies used the PHQ-9, 26% (10/38) used the BDI-II, 16% (6/38) used the DASS-21, 11% (4/38) used the CES-D, 3% (1/38) used the HADS, 3% (1/38) used the IDS-SR, 3% (1/38) used the CCAPS-34, and 3% (1/38) used the PROMIS.

Of the 50 different comparisons used in the 38 different studies, 71% (27/38) contained gamification elements and 29% (11/38) did not. The number of gamification elements observed in each study are shown in Multimedia Appendix 5. Table 1 shows the type of intervention, app, and number of gamification elements for each article. Multimedia Appendix 6 reports supplemental meta-analytic results for long-term follow-up time points and control variables.

### Primary Analysis

**Figure 3:**
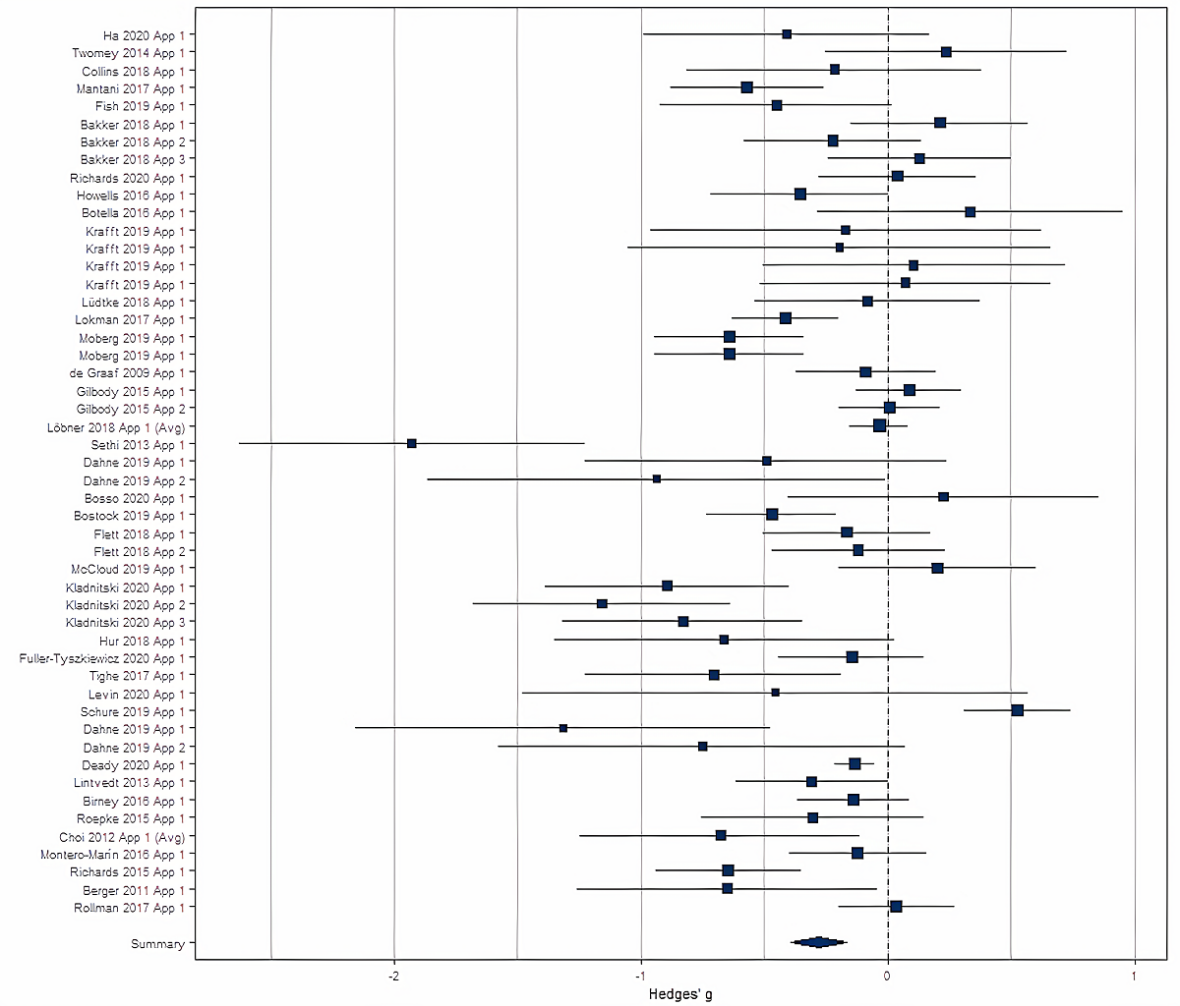
Forest plot for all studies (n=50 comparisons) showing the effect sizes for each. Author and year for each study are listed on the y-axis.

A forest plot for the postintervention differences between the mental health app intervention group and the control group is shown in Figure 3. The random effects model for all eligible studies (n=50 comparisons) revealed a small to medium effect of mental health apps in reducing depressive symptoms compared to controls (g=–0.27, 95% CI –0.36 to –0.17; P<.001). However, significant heterogeneity in the results were observed (I^2^=0.76, τ^2^=0.076; P<.001). The gamification moderator was not a significant predictor of depressive symptoms (β=–0.03, SE=0.04; P=.38); the intervention duration moderator was also not a significant predictor (β=–0.02, SE=0.04; P=.67).

### Sensitivity Analysis: CBT-Only Studies

A sensitivity analysis was performed for app comparisons that involved CBT-based therapy (37/50, 74%). The overall effect size was similar to the overall analysis (g=–0.30, 95% CI –0.42 to –0.17; P<.001), and there was no significant effect of gamification elements as a moderator (β=–0.04, SE=0.04; P=.31) or of study duration as a moderator (β=–0.02, SE=0.05; P=.69). Multimedia Appendix 7 show the funnel plot for this analysis.

### Secondary Analysis: Adherence

The secondary analysis examining comparisons that included a measure of adherence (36/50, 72%) failed to show a significant effect of gamification elements (β=–1.93, SE=2.28; P=.40) on adherence rates. However, intervention duration was a significant predictor such that longer interventions were associated with higher adherence rates (β=11.33, SE=3.57; P=.003). Similarly, when examining only the CBT-based mental health apps (25/50, 50%), there was no effect of gamification elements on adherence (β=0.17, SE=2.51; P=.95), but intervention duration positively predicted adherence (β=12.23, SE=4.21; P=.008).

### Funnel Plot Results

**Figure 4:**
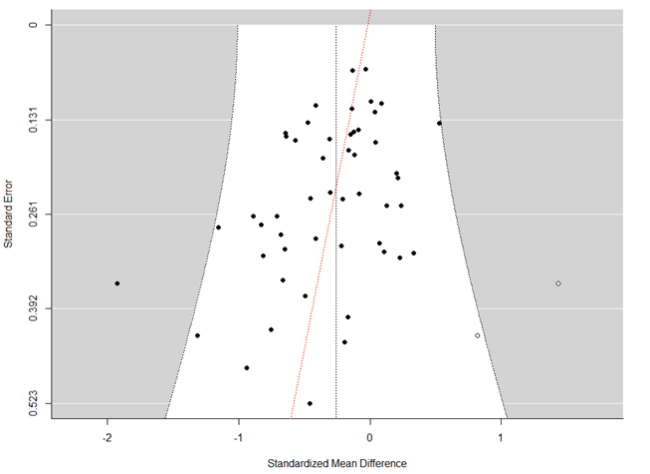
Funnel plot for all studies (n=50 comparisons) showing the heterogeneity for each.

Examination of the funnel plot of the posttreatment effect SEs indicated heteroscedasticity. The Egger test of asymmetry was significant (Q_50_=214.46, P<.001). A trim-and-fill analysis suggested a trending finding of three missing publications to the right, though this did not reach the level of statistical significance (β=2.45, P=.13). To be conservative, the pseudo-CIs in Figure 4 were adjusted per τ^2^, with trim-and-fill studies as white dots. Controlling for the violations of these assumptions, the overall random effects model was significant (β=–0.26, SE=0.06; P<.001), indicating a significant improvement overall.

## Discussion

### Principal Findings

This meta-analysis provides a comprehensive update about the effectiveness of mental health apps in reducing depressive symptoms, and it tests whether mental health apps with gamification elements are effective for reducing depressive symptoms. Results indicated that mental health apps are effective for reducing depressive symptoms, but gamification elements within mental health apps do not seem to reduce depressive symptoms or increase the adherence of using mental health apps.

Examination of mental health apps that exclusively employed computerized CBT (excluding mindfulness and ACT) similarly failed to show an effect of gamification on depressive symptom reduction. It should be noted that some of the included studies had a high risk of bias and, as such, the results should be interpreted with caution. Nevertheless, the evidence from this meta-analysis suggests that CBT effectively aids with the control, maintenance, and reduction of depressive symptoms, but gamification and reward elements embedded in mental health apps do not significantly alleviate such symptoms.

Consistent with prior systematic reviews and meta-analyses on digital interventions for depression, we found that mental health app interventions are moderately effective in reducing depressive symptoms compared to controls [26,37,38,41,89]. Effect sizes from these prior meta-analyses ranged from 0.33 to 0.58, which is slightly higher than the small to moderate effect size observed in this meta-analysis. While this research provides strong consensus of a general positive effect of mental health apps on alleviating depressive symptoms, the specific elements that contribute to this effect remain largely elusive. Previous systematic reviews have demonstrated that guidance and support from a professional can augment the effectiveness of mental health app interventions [37,105]. Other work suggests that incorporating reminders into digital mental health interventions can promote engagement and adherence with the intervention, which may, in turn, enhance the intervention’s therapeutic benefits [38]. However, the results of this meta-analysis did not show a significant relationship between gamification and adherence rates. Understanding how such features work together to mitigate depressive symptoms is critical for improving mental health app development.

One potential explanation of why gamification elements do not moderate depressive symptoms may be reward sensitivity. Depression generally has been associated with hyposensitivity to rewards, particularly among those with anhedonic symptoms [11-13,16,17]. This diminished reward-related neural activity may decrease motivation to obtain rewards or diminish the positive reward experience. While the UGM model proposes reward as a critical part of motivation to ensure engagement, it also suggests moderating effects, such as self-efficacy and locus of control. These variables have known negative relationships with depression [106,107] and may make gamification less effective. An alternative explanation for the results may be that gamification does not engender additive benefits. The included mental health apps all used strong, evidence-based therapeutic interventions, including CBT, ACT, and mindfulness. Based on the results of this meta-analysis, these interventions appear to be sufficient in mitigating depressive symptoms.

Overall, 30 studies in this meta-analysis showed a significant effect, while nine studies failed to produce significant reductions in depressive symptoms. In terms of gamification elements, four of the apps that were associated with nonsignificant findings included at least two elements of gamification: Kokoro, Headspace, MoodGYM, and Be Good to Yourself [50,52,59,67]. In contrast, two of these apps—Headspace and MoodGYM—were used in other studies, where they produced significant positive changes in depressive symptoms [31,53,64,65,68,69]. These mixed findings support the notion that while mental health apps may have a future in telemedicine and psychological settings [89,108], more research is needed to understand which mental health app features are integral to improving mental health symptoms. From there, these facets can be properly implemented as a methodologically reliable therapeutic technique.

The push for the creation of efficient and scientifically supported mental health apps grows each year as technology becomes more ubiquitous [109]. These apps could aid therapists who are unable to accept any new clients and people who may not have access to psychological centers or counselors due to financial strains, location, or disabilities. This meta-analysis adds to the current literature by suggesting against overreliance on reward and gamification elements as major reducers of depressive symptoms. These elements may be beneficial in mental health apps, but no more so than other evidence-based therapeutic features. Many of the mental health apps currently available to the general public lack valid testing of their efficacy; thus, there is a strong need for rigorous evaluation of such apps on psychological outcomes. Indeed, in this meta-analysis, nearly half of the included studies incorporated gamification elements, yet the results suggest that such elements exert minimal therapeutic benefits. Significantly more research is needed to identify which specific mental health app features maximize therapeutic effects.

### Limitations

This systematic review and meta-analysis had some limitations, which were largely related to inadequacies in the studies available for analyses. Results were calculated based on aggregated samples, which may have caused a certain level of ecological bias. Due to the disparate sample of apps, heterogeneity was detected in all primary analyses. However, heterogeneity was nearly identical to past research on this topic, and we took statistical efforts to minimize this impact [38]. In addition, the meta-analysis was not exclusive to individuals with clinical levels of depression, but all experiencing depressive symptoms. No data sets were consistently available across all studies to assess clinical depression diagnoses or psychiatric comorbidities. Consequently, the findings may not generalize to individuals with severe depression or individuals with other mental health conditions.

### Future Research Using Gamification Elements

The main findings of this study were that mental health apps are effective for reducing depressive symptoms, but gamification elements within these mental health apps do not seem to affect depressive symptoms. It is possible that mental health apps with gamification elements may influence patients managing anxiety, stress, or other conditions where anhedonia is not present. Thus, gamification may not be a promising app feature for depressive symptoms but may hold promise for other mental health conditions. Future research should consider examining the effectiveness of mental health apps with gamification on other mental health conditions. While previous research has investigated the effectiveness of mental health apps on anxiety and life satisfaction [50,56], the effect of gamification elements on these psychological factors remains underexplored. Moreover, there is a need for additional research to better characterize the usability benefits and user preferences of mental health apps. If gamification within mental health apps is not effective for individuals experiencing depressive symptoms, then it is important to identify other potential features. Designing specific features that may motivate users with depression toward continued mental health app adherence could lead to beneficial outcomes.

### Conclusions

Mental health apps have proven to be a useful tool in reducing depressive symptoms with or without the inclusion of gamification elements. These results demonstrate that although there is a significant improvement in using mental health apps overall, there is no evidence to suggest that gamification makes outcomes significantly better or worse. Additional elements, such as personalization, motivational reminders, social support, and usability, need to be investigated. Mental health apps may provide a readily available option for global psychological care; however, supplementary research is needed on their effectiveness before reliable implementation into the health care system can occur. 

## Appendix

Multimedia Appendix 1 Search terms for the meta-analysis.

Multimedia Appendix 2 Database screening tool.

Multimedia Appendix 3 Study demographics extracted from articles: sample, length, population, age, and gender.

Multimedia Appendix 4 Quality assessment screening.

Multimedia Appendix 5 Gamification screening for publications.

Multimedia Appendix 6 Sensitivity and supplemental analyses.

Multimedia Appendix 7 Sensitivity analysis for cognitive behavioral therapy (CBT)–only studies.

## Abbreviations

ACT: acceptance and commitment therapy
BA: behavioral activation
BDI-II: Beck Depression Inventory-II
CBT: cognitive behavioral therapy
CCAPS-34: Counseling Center Assessment of Psychological Symptoms-34
CES-D: Center for Epidemiological Studies Depression Scale
DASS-21: 21-item Depression, Anxiety, and Stress Scale
HADS: Hospital Anxiety and Depression Scale
IDS-SR: Inventory of Depressive Symptomatology Self-Report
PHQ-9: 9-item Patient Health Questionnaire
PRISMA: Preferred Reporting Items for Systematic Reviews and Meta-Analyses
PROMIS: Patient-Reported Outcomes Measurement Information System
RCT: randomized controlled trial
UGM: Unified Gamification and Motivation
