# Supplementary material for: Examining the Effectiveness of Gamification in Mental Health Apps for Depression: Systematic Review and Meta-analysis
Source: JMIR Ment Health. 2021 Nov 29;8(11):e32199. doi: 10.2196/32199 (PMC8669581; doi:10.2196/32199)
Supplement: Multimedia Appendix 1 [file mental_v8i11e32199_app1.docx]

**Multimedia Appendix 1: Search Terms for Meta-Analysis**

| Table S1. |  |
| --- | --- |
| **Concept 1: App-Based** |  |
| “Mental Health App*” | “MHapp” |
| “App-based” | “Self-help app” |
| “e-health” | “CCBT” |
| “CBT+App” | “Mindfulness + app” |
| “Game-based treatment + app” | “Relaxation + app” |
| “Acceptance and Commitment Therapy” | “Meditation+ app” |
| **Concept 2: Mental Health** |  |
| “Depression” |  |
| **Concept 3: Gamification** |  |
| “Gamification” | “Game” |
| “Reward” | “Points” |
| “Money” | “Monetary” |
| “Feedback” | “Reinforcement” |

*Note*: Search terms were categorized into three concepts: App-based, Mental

Health, and Gamification. Within each concept (e.g., App-Based), multiple tags

were identified that reflected this concept (e.g., Mental Health App, MHapp.)

Search terms consisted of all possible pairwise combinations of tags within one

concept to the other two concepts’ tags.
