# Supplementary material for: Examining the Effectiveness of Gamification in Mental Health Apps for Depression: Systematic Review and Meta-analysis
Source: JMIR Ment Health. 2021 Nov 29;8(11):e32199. doi: 10.2196/32199 (PMC8669581; doi:10.2196/32199)
Supplement: Multimedia Appendix 2 [file mental_v8i11e32199_app2.docx]

**Multimedia Appendix 2: Database Screening Tool**

A total list of 14 questions for screening each article was created for more rapid and meticulous process. These 14 questions were derived from the inclusion and exclusion criteria with the first 5 meant for screening the title and abstract and the remaining 9 meant for screening the full articles.

**Title and Abstract Screening**

1. Does the **citation** indicate publication before 2005 or after 2021?

a. Yes: stop screening

b. No: continue screening

2. Does the **title or abstract** use English?

a. Yes: continue screening

b. No: stop screening

3. Does the **title or abstract** indicate a review, meta-analysis, care study, opinion piece, reply, or portion of a book?

a. Yes: stop screening

b. No: continue screening

4. Does the **title or abstract** relate to mental health?

a. Yes: continue screening

b. No: stop screening

5. Is the **title or abstract** the same as a previous article (duplicate)?

a. Yes: Stop screening – exclude

b. No: Continue screening

**Article Screening**

6. Does the **article** include non-human participants?

a. No or Unsure/Unclear: continue screening

b. Yes: stop screening

7. Does the **article** indicate CBT or ACT intervention, meditation, mindfulness, and relaxation as part of a tech-based intervention?

a. Yes or Unsure/Unclear: continue screening

b. No: stop screening

8. Does the **article** indicate a control group/experimental/quasi-experimental design?

a. Yes: continue screening

b. No: stop screening

9. Does the **article** relate to the research question?

a. Yes or Unsure/Unclear: continue screening

b. No: stop screening

10. Does the **article** include participants younger than 18?

a. No or Unsure/Unclear: continue screening

b. Yes: stop screening

11. Does the **article** include participants with life threatening or terminal conditions?

a. No or Unsure/Unclear: continue screening

b. Yes: stop screening

12. Does the **article** include a measure of depression?

a. Yes: Continue screening

b. No: Stop screening – Exclude

13. Does the **article** have missing data?

a. Yes: Stop screening – exclude

b. No: Continue screening

14. Does the **article** include therapist-supported interventions?

a. Yes: Stop screening – exclude

b. No: Continue screening

**Decision**: **Should this article be included?**

a. **Yes**, all 14 screening questions answered Yes or Unclear

b. **No**, at least one answers definitely “No”
