# Supplementary material for: Examining the Effectiveness of Gamification in Mental Health Apps for Depression: Systematic Review and Meta-analysis
Source: JMIR Ment Health. 2021 Nov 29;8(11):e32199. doi: 10.2196/32199 (PMC8669581; doi:10.2196/32199)
Supplement: Multimedia Appendix 3 [file mental_v8i11e32199_app3.docx]

**Multimedia Appendix 3**

### Study demographics: sample, length, population, age, and gender.

| **First Author Last Name** | **Publication Date** | **n** | **Base Line Sample** | **Study Length** | **Population** | **Age** | **Gender** |
| --- | --- | --- | --- | --- | --- | --- | --- |
| Bakker | 2018 | n = 141 | MoodPrism = 26 MoodMission=23 MoodKit = 39 Control = 53 | 30 days | Adults | MoodPrism = 36.1,  MoodMission = 33.3,  MoodKit = 33.8,  Control = 33.6 | MoodPrism (F = 89%),  MoodMission  (F = 82%),  MoodKit (F = 75%),  Control (F = 77%) |
| Birney | 2016 | n = 300 | Intervention = 150 Control = 150 | 10 weeks | Adults with depressive symptoms | Intervention = 40.6  Control = 40.7 | Intervention  (F = 74.6%) &  Control (F = 78.7%) |
| Berger | 2011 | n = 51 | Intervention = 25  Control = 26 | 10 weeks | Adults with depression | Intervention = 38.6  Control = 39.6 | Intervention  (F = 72%)  Control  (F = 69.2%) |
| Bosso | 2020 | n = 44 | Intervention = 22 Control = 22 | 5 weeks | College students | Intervention = 24.14  Control = 23.59 | Intervention & Control (F = 90.9%) |
| Bostock | 2019 | n = 238 | Intervention = 128 Control = 110 | 8 weeks | Workers from UK companies | Combined Sample  = 35.5 | Intervention & Control (F = 59.2%) |
| Botella | 2016 | n = 60 | Intervention = 22 Sensor=19  Control = 19 | 10 weeks | Unemployed men | Combined Sample  = 32.25 | Intervention Control (F = 0%) |
| Choi | 2012 | n = 63 | Intervention = 32 Control = 31 | 8 weeks | Adults with depression | Intervention = 40.6  Control = 37.8 | Intervention  (F = 88%)  Control  (F =73%) |
| Collins | 2018 | n = 79 | Intervention = 60  Control = 19 | 28 days | Adults referred to psychological services | Intervention = 35.32  Control = 38.32 | Intervention  (F = 76%)  Control (F = 68.4%) |
| Dahne | 2019 | n = 42 | Aptívate = 22 iCouch CBT = 9 Control = 11 | 8 weeks | Adults | Aptívate = 32.68,  iCouch CBT = 40.56,  Control = 39.09 | Aptívate  (F = 77.3%),  iCouch CBT  (F = 44.4%),  Control ( F= 63.6%) |
| Dahne | 2019 | n = 52 | Moodivate = 24 Moodkit = 19 Control = 9 | 8 weeks | Adults with depressive symptoms | Moodivate = 44.67,  MoodKit = 43.00, &  Control = 43.11 | Moodivate  (F = 83.3%),  MoodKit  (F = 78.9%),  Control (F = 84.6%) |
| Deady | 2020 | n=2271 | Intervention=1128 Control = 1143 | 30-day | Adult with non-clinical depressive symptoms | Intervention = 40.20  Control = 40.32 | Intervention  (F = 27.6%)  Control (F = 24.0%) |
| de Graaf | 2009 | n = 203 | Intervention = 100 Control = 103 | 2 months | Adults with depressive symptoms | Intervention = 44.3  & Control = 45.1 | Intervention  (F = 52%) & Control  (F = 55.3%) |
| Fish | 2019 | n = 72 | Intervention = 33 Control = 39 | 2 weeks | College students | Combined Sample  = 21 | Intervention & Control (F = 96%) |
| Flett | 2018 | n = 208 | Headspace = 72 Smiling Mind= 63 Control = 73 | 10 days | College Students | Combined Sample  = 20.8 | No information |
| Fuller-Tyszkiewicz | 2020 | n = 183 | Intervention = 73 Control = 110 | 5 weeks | Caregivers | Intervention = 40.29  Control = 39.21 | Intervention  (F = 95%)  Control  (F = 95.4%) |
| Gilbody | 2015 | n= 691 | BTB = 210 MoodGYM = 242 Control = 239 | 4 months | Adults with depressive symptoms | Beating the Blues = 39.61,  MoodGYM = 39.43, Control = 40.52 | Beating the Blues  (F = 68%)  MoodGYM  (F = 65%),  Control  (F = 68%) |
| Ha | 2020 | n = 47 | Intervention = 25  Control = 22 | 2 weeks | College students | Intervention = 21.00  Control = 21.95 | Intervention (F = 68%)  Control (F = 73%) |
| Howells | 2016 | n = 121 | Intervention = 57 Control = 64 | 10 days | Adults seeking happiness | Intervention = 39.74  Control = 40.86 | Intervention  (F = 85.6%)  Control (F = 90.7%) |
| Hur | 2018 | n = 48 | Intervention = 24 Control = 24 | 3 weeks | Adults with depression | Intervention = 24.75  Control = 22.65 | Combined Sample (F = 86.67%) |
| Kladnitski | 2020 | n = 158 | iCBT = 39  iMT = 40 MEiCBT = 40 Control = 39 | 14 weeks | Adults with depression or anxiety | iCBT = 36.69, iMT = 37.10, MEiCBT = 41.38, &  Control 41.69 | iCBT (F = 84.6%), iMT (F = 85%), MEiCBT (F = 90%),  & Control =  (F = 84.6%) |
| Krafft | 2019 | n = 98 | Simple Intervention = 33 Complex Intervention = 34 Control=31 | 4 weeks | College students and individuals who wanted help | No information | No information |
| Levin | 2020 | n = 23 | Intervention = 10 Control = 13 | 4 weeks | College students | Intervention = 19.90  Control = 20.85 | Combined Sample (F = 100%) |
| Lintvedt | 2013 | n = 163 | Intervention = 81 Control = 82 | 2 months | College students | Intervention = 28.8  Control = 27.5 | Intervention  (F =67.9%) &  Control (F = 85.4%) |
| Löbner | 2018 | n = 112 | Intervention = 65 Control = 47 | 6 weeks | Adults with depressive symptoms | Intervention = 40.2  Control = 47.5 | Intervention (F = 69%)  Control (F = 67.9) |
| Lokman | 2017 | n = 329 | Intervention = 165 Control = 164 | 4 weeks | Adults with depressive symptoms | Intervention = 42.85  Control = 43.65 | Intervention  (F = 73.9%)  & Control  (F = 77.4%) |
| Lüdtke | 2018 | n = 90 | Intervention = 45 Control = 45 | 4 weeks | Adults who wanted help with depression | Intervention = 44.57  Control = 41.20 | Intervention  (F = 75%)  Control (F=81.8%) |
| Mantani | 2017 | n = 164 | Intervention = 81 Control = 83 | 9 weeks | Adults with depression | Intervention = 40.2  Control = 41.6 | Intervention  (F = 57%)  Control (F = 50%) |
| McCloud | 2019 | n = 168 | Intervention = 84 Control = 84 | 6 weeks | College Students | Intervention = 25.1  & Control = 23.5 | Intervention  (F = 82%)  Control (F = 88%) |
| Moberg | 2019 | n = 500 | Intervention = 253 Control = 247 | 1-month | Adults with anxiety or depression | Combined Sample  = 30.2 | Intervention  (F = 75%)  Control (F = 74%) |
| Montero-Marín | 2016 | n = 200 | Intervention = 98 Control = 102 | 3 months | Adults with depression | Intervention = 42.57  Control = 43.06 | Intervention  (F = 73.5%)  Control  (F = 74.5%) |
| Richards | 2020 | n = 361 | Intervention = 241 Control = 120 | 8 weeks | New IAPT referrals | No information | Intervention  (F = 71.8%)  Control = (70.8%) |
| Richards | 2015 | n = 188 | Intervention = 96 Control = 92 | 6 months | Adults with depressive symptoms | Intervention = 40.63  Control = 39.05 | Intervention  (F = 74%)  Control  (F = 71.7%) |
| Roepke | 2015 | n = 186 | Intervention = 3 Control = 93 | 1 month | Adults with depression | Intervention = 42.28  Control = 40.27 | Intervention  (F = 61.29%)  Control (F=76.34%) |
| Rollman | 2017 | n = 402 | Intervention = 301 Control = 101 | 6 months | Adults seeking mental health treatment | Intervention = 43.0  Control = 41.7 | Intervention  (F = 78.1%)  Control  (F = 81.2%) |
| Schure | 2019 | n = 343 | Intervention = 181 Control = 162 | 8 weeks | Adults with depressive symptoms | Intervention = 42.1  Control = 43.8 | Intervention  (F = 88.4%)  Control  (F = 81.2%) |
| Sethi | 2013 | n = 46 | Intervention = 23 Control = 23 | 5 weeks | Adults age 18-25 from community centers | Intervention = 20.78  Control = 19.47 | Intervention  (F = 82%) &  Control (F = 57%) |
| Tighe | 2017 | n = 61 | Intervention = 31 Control = 30 | 6 weeks | Adults age 18-35 | Intervention = 27.48  Control = 24.97 | Intervention  (F = 65%)  Control (F = 63%) |
| Twomey | 2014 | n = 149 | Intervention = 80 Control = 69 | 32 days | Adult public mental health users | Intervention = 37.29  Control = 32.61 | Intervention  (F = 89.3%)  Control (F = 68.4%) |

*^Note: F= Females^*
