# Supplementary material for: Examining the Effectiveness of Gamification in Mental Health Apps for Depression: Systematic Review and Meta-analysis
Source: JMIR Ment Health. 2021 Nov 29;8(11):e32199. doi: 10.2196/32199 (PMC8669581; doi:10.2196/32199)
Supplement: Multimedia Appendix 4 [file mental_v8i11e32199_app4.docx]

**Multimedia Appendix 4: Quality Assessment Screening Table**

| **First Author Name** | **Year** | **Random Sequence Generation** | **Allocation Conceal-ment** | **Blinding of outcome** | **Blinding personnel & users** | **Incomplete Outcome Data** | **Selective Outcome Reporting** | **Other Bias** | **Total** |
| --- | --- | --- | --- | --- | --- | --- | --- | --- | --- |
| Bakker | 2018 | 0 | 0 | 0 | 1 | 0 | 0 | 1 | 2 |
| Berger | 2011 | 1 | 0 | 1 | 1 | 0 | 0 | 1 | 4 |
| Birney | 2016 | 1 | 0 | 1 | 1 | 0 | 1 | 0 | 4 |
| Bosso | 2020 | 1 | 0 | 1 | 1 | 0 | 0 | 1 | 4 |
| Bostock | 2019 | 1 | 0 | 1 | 1 | 0 | 0 | 1 | 4 |
| Botella | 2016 | 1 | 0 | 1 | 1 | 0 | 0 | 1 | 4 |
| Choi | 2012 | 0 | 0 | 1 | 2 | 0 | 2 | 2 | 7 |
| Collins | 2018 | NA | 2 | 1 | 2 | 0 | 0 | 2 | 7 |
| Dahne | 2019 | 1 | 0 | 1 | 1 | 1 | 0 | 0 | 4 |
| Dahne | 2019 | 1 | 0 | 1 | 1 | 1 | 0 | 0 | 4 |
| Deady | 2020 | 0 | 0 | 0 | 0 | 0 | 2 | 0 | 2 |
| de Graaf | 2009 | 1 | 0 | 1 | 1 | 0 | 1 | 0 | 3 |
|  |  |  |  |  |  |  |  |  |  |
| Fish | 2019 | 0 | 0 | 1 | 1 | 0 | 0 | 0 | 2 |
| Flett | 2018 | 0 | 0 | 1 | 1 | 1 | 0 | 1 | 4 |
| Fuller-Tyszkiewicz | 2020 | 0 | 0 | 2 | 0 | 1 | 2 | 2 | 7 |
| Gilbody | 2015 | 0 | 0 | 0 | 0 | 0 | 0 | 1 | 1 |
| Ha | 2020 | 0 | 0 | 1 | 2 | 0 | 0 | 0 | 3 |
| Howells | 2016 | 0 | 0 | 0 | 0 | 1 | 0 | 1 | 2 |
| Hur | 2018 | 0 | 0 | 0 | 1 | 1 | 0 | 0 | 2 |
| Kladnitski | 2020 | 1 | 0 | 1 | 0 | 0 | 2 | 0 | 4 |
| Krafft | 2019 | 1 | 0 | 1 | 1 | 0 | 0 | 1 | 3 |
| Levin | 2020 | 0 | 0 | 1 | 1 | 0 | 0 | 0 | 2 |
| Lintvedt | 2013 | 0 | 0 | 1 | 1 | 1 | 0 | 1 | 4 |
| Löbner | 2018 | 0 | 0 | 2 | 2 | 0 | 0 | 1 | 5 |
| Lokman | 2017 | 0 | 0 | 2 | 2 | 0 | 0 | 1 | 5 |
| Lüdtke | 2018 | 0 | 0 | 2 | 1 | 0 | 1 | 0 | 4 |
| Mantani | 2017 | 0 | 0 | 2 | 0 | 1 | 0 | 1 | 4 |
| McCloud | 2019 | 0 | 0 | 2 | 2 | 0 | 0 | 1 | 5 |
| Moberg | 2019 | 1 | 0 | 1 | 1 | 1 | 2 | 1 | 7 |
| Montero-Marín | 2016 | 0 | 0 | 2 | 0 | 1 | 0 | 0 | 3 |
| Richards | 2015 | 0 | 0 | 1 | 1 | 0 | 2 | 0 | 4 |
| Richards | 2020 | 0 | 0 | 0 | 2 | 0 | 0 | 1 | 3 |
| Roepke | 2015 | 0 | 0 | 0 | 1 | 1 | 0 | 2 | 4 |
| Rollman | 2017 | 0 | 0 | 0 | 2 | 0 | 0 | 0 | 2 |
| Schure | 2019 | 1 | 0 | 0 | 1 | 0 | 2 | 0 | 4 |
| Sethi | 2013 | 0 | 0 | 1 | 1 | 0 | 0 | 0 | 2 |
| Tighe | 2017 | 2 | 0 | 1 | 2 | 1 | 0 | 0 | 6 |
| Twomey | 2014 | 0 | 0 | 2 | 2 | 1 | 0 | 2 | 7 |
