# Supplementary material for: Examining the Effectiveness of Gamification in Mental Health Apps for Depression: Systematic Review and Meta-analysis
Source: JMIR Ment Health. 2021 Nov 29;8(11):e32199. doi: 10.2196/32199 (PMC8669581; doi:10.2196/32199)
Supplement: Multimedia Appendix 5 [file mental_v8i11e32199_app5.docx]

### **Multimedia Appendix 5: Gamification Screening for the Publications**

| **Author/ Year** | **App** | **Reward** | **Challenge/Game** | **Story** | **Feed-back** | **Clear Goals** | **Levels** | **Points** | **Achieve-ment; Badges** | **None** |
| --- | --- | --- | --- | --- | --- | --- | --- | --- | --- | --- |
| Bakker, et al., 2018 | MoodPrism | X |  |  | X |  |  |  | X |  |
| Bakker, et al., 2018 | MoodMission | X |  |  |  | X |  |  | X |  |
| Bakker, et al., 2018 | MoodKit |  |  |  |  |  |  |  |  | X |
| Berger, et al., 2011 | Deprexis |  |  |  | X |  |  |  |  |  |
| Birney, et al., 2016 | Moodhacker |  |  |  |  |  |  |  |  | X |
| Bosso, et al., 2020 | Headspace | X |  |  |  |  | X | X |  |  |
| Bostock, et al., 2019 | Headspace | X |  |  |  |  | X | X |  |  |
| Botella, et al., 2016 | Smiling is Fun |  |  |  | X |  |  |  |  |  |
| Choi, et al., 2012 | Brighten Your Mood |  |  |  |  |  |  |  |  | X |
| Collins, 2018 | MindWise |  |  |  |  |  |  |  |  | X |
| Dahne, et al., 2019 | Aptivate |  |  |  | X |  |  |  | X |  |
| Dahne, et al., 2019 | iCouch CBT |  |  |  |  |  |  |  |  | X |
| Dahne, et al., 2019 | Moodivate |  |  |  | X |  |  |  | X |  |
| Dahne, et al., 2019 | Moodkit |  |  |  |  |  |  |  |  | X |
| Deady, et al., 2020 | Headgear |  | X |  | X |  |  |  |  |  |
| de Graaf, et al., 2009 | Colour Your Life |  |  |  |  |  |  |  |  | X |
| Fish, et al., 2019 | Headspace | X |  |  |  |  |  | X | X |  |
| Flett, et al., 2018 | Headspace | X |  |  |  |  | X | X |  |  |
| Flett, et al., 2018 | Smiling Mind | X |  |  |  |  |  |  |  |  |
| Flett, et al., 2018 | Evernote |  |  |  |  |  |  |  |  | X |
| Fuller-Tyszkiewicz, et al., 2020 | StressLess |  |  |  | X |  |  |  |  |  |
| Fuller-Tyszkiewicz, et al., 2020 | StressMonitor |  |  |  | X |  |  |  |  |  |
| Gilbody, et al., 2015 | Beat the Blues |  |  |  |  |  |  |  |  | X |
| Gilbody, et al., 2015 | MoodGYM |  | X |  | X |  |  |  |  |  |
| Ha, 2020 | Spring | X |  |  | X |  |  |  |  |  |
| Howells, et al., 2016 | Catch Notes |  |  |  |  |  |  |  |  | X |
| Howells, et al., 2016 | HeadSpace | X |  |  |  |  | X | X |  |  |
| Hur, et al., 2018 | Todac Todac |  |  |  | X |  |  |  |  |  |
| Kladnitski, et al., 2020 | Virtual Clinic iCBT program | X |  | X |  |  |  |  |  |  |
| Kladnitski, et al., 2020 | Virtual Clinic MEiCBT program | X |  | X |  |  |  |  |  |  |
| Kladnitski, et al., 2020 | Virtual Clinic iMT program | X |  | X |  |  |  |  |  |  |
| Kraft, et al., 2019 | Simple Matrix App |  |  |  | X |  |  |  |  |  |
| Kraft, et al., 2019 | Complex Matrix App |  |  |  | X | X |  |  |  |  |
| Levin, et al., 2017 | Stop, Breathe, Think |  |  |  |  |  |  |  |  | X |
| Lintvedt, et al., 2013 | MoodGYM |  | X |  | X |  |  |  |  |  |
| Lintvedt, et al., 2013 | Blue Pages |  |  |  |  |  |  |  |  | X |
| Löbner, et al., 2018 | MoodGYM |  | X |  | X |  |  |  |  |  |
| Lokman, et al., 2017 | CDMI Sleep Better, Worry Less, Stress Less |  |  |  | X |  |  |  |  |  |
| Lüdtke, et al., 2018 | Be Good to Yourself |  | X |  | X | X |  |  |  |  |
| Mantani, et al., 2017 | Kokoro | X | X |  | X | X | X | X |  |  |
| McCloud, et al., 2019 | Feel Stress Free |  | X |  | X |  |  |  |  |  |
| Moberg, et al., 2019 | Pacifica |  | X |  | X | X |  |  |  |  |
| Montero-Marín et al., 2016 | Smiling is Fun |  |  | X |  |  |  |  |  |  |
| Richards, et al., 2020 | Space from Depression |  | X |  | X |  |  |  |  |  |
| Richards, et al., 2015 | Space from Depression |  | X |  | X |  |  |  |  |  |
| Roepke, et al., 2015 | SuperBetter |  | X | X | X | X | X | X | X |  |
| Rollman et al., 2017 | Beating the Blues |  |  |  |  |  |  |  |  | X |
| Schure, et al., 2019 | Thrive |  |  | X | X |  |  |  | X |  |
| Sethi, et al., 2013 | MoodGYM |  | X |  | X |  |  |  |  |  |
| Tighe, et al., 2017 | iBobbly |  |  |  |  |  |  |  |  | X |
| Twomey, et al., 2014 | MoodGYM |  | X |  | X |  |  |  |  |  |

*Note*: Gamification screening for the 37 different apps.
