# Supplementary material for: Examining the Effectiveness of Gamification in Mental Health Apps for Depression: Systematic Review and Meta-analysis
Source: JMIR Ment Health. 2021 Nov 29;8(11):e32199. doi: 10.2196/32199 (PMC8669581; doi:10.2196/32199)
Supplement: Multimedia Appendix 6 [file mental_v8i11e32199_app6.docx]

### **Multimedia Appendix 6**

### Secondary Analysis: Follow-up Time Points

A secondary analysis using a random effects model with the gamification moderator was performed for studies that included a follow-up timepoint that extended beyond the intervention period. The follow-up analysis included 11 studies with 16 comparisons (2,381 intervention participants and 2,553 control participants). The effect of mental health applications on depressive symptoms was small at long-term follow-up timepoints [g= -.15 (95% CI: -0.22; -0.08), *P*<.05], and gamification did not significantly influence this effect (*P*=.61). Effect sizes for mental health applications with gamification was small but significant [g= -.16 (95% CI: -0.24; -0.08), *P*<.05], while mental health applications without gamification [g= -.13 (95% CI: -0.29; 0.02)] did not have a significant effect on depressive symptoms as follow-up. The Egger test of asymmetry was non-significant (Q(10)=6.35, *p*=.79).

### Secondary Analysis: Adherence

A meta-regression analysis examining the interaction between adherence rates and number of gamification elements on post-intervention depressive symptom scores failed to show a significant interaction (*P*=.68). A similar meta-regression examining exclusively CBT-based mental applications also showed no significant interaction between adherence and gamification elements (*P*=.99).

### Control Variables

Supplemental analyses for depressive symptoms scores and adherence rates were performed to control for age, gender, and pre-treatment depression levels. Predictors included number of gamification elements and intervention duration (in months). Seven studies had missing values. For depressive symptom scores in the intervention condition, number of gamification elements (β=-0.03, SE=.04, *P=.*51), study duration (β=-.01, SE=.05, *P=.*91), age (β=.01, SE=.01, *P=.*15), gender (β=.02, SE=.01, *P=.*80) and pre-treatment depressive symptom scores (β=-.01, SE=.01, *P*=.35) were all nonsignificant.

For adherence rates, number of gamification elements (β=-0.61, SE=1.04, *P=.*56), age (β=-.21, SE=.13, *P=.*11), gender (β=-.08, SE=.13, *P=.*59) and pre-treatment depressive symptom scores (β=.02, SE=.10, *P*=.99) were non-significant. Intervention duration was a marginally significant predictor of adherence rates in the intervention condition (β=3.04 SE=1.52, *P=.*051) such that longer duration predicted higher adherence rates.
