# Supplementary material for: Examining the Effectiveness of Gamification in Mental Health Apps for Depression: Systematic Review and Meta-analysis
Source: JMIR Ment Health. 2021 Nov 29;8(11):e32199. doi: 10.2196/32199 (PMC8669581; doi:10.2196/32199)

**Multimedia Appendix 7: Sensitivity Analysis: CBT only Therapy**

The sensitivity analysis with CBT-based apps revealed a small-to-medium effect size on depressive symptoms [g=-0.30 (95% CI: -0.42; -0.17) *P*<.001], and the gamification moderator (*P=*.31) and intervention duration moderator (*P=*.68) were not significant.

Examination of the funnel plot (Figure S1) indicated heteroscedasticity. The Egger test of asymmetry was significant (Q(36)=193.40, *P*<.001). The trim-and-fill analysis of CBT only interventions was identical to the full sample, SE = 2.45, *P*=.13 To be conservative, Figure S1's pseudo-confidence intervals were adjusted per tau-squared, with trim-and-filled studies as white dots. The overall random-effects model was significant (β = -.27, SE =.08, *P*<.001), indicating a significant improvement overall.

### *Figure S1*. Funnel plot for all CBT-based studies (n = 37).


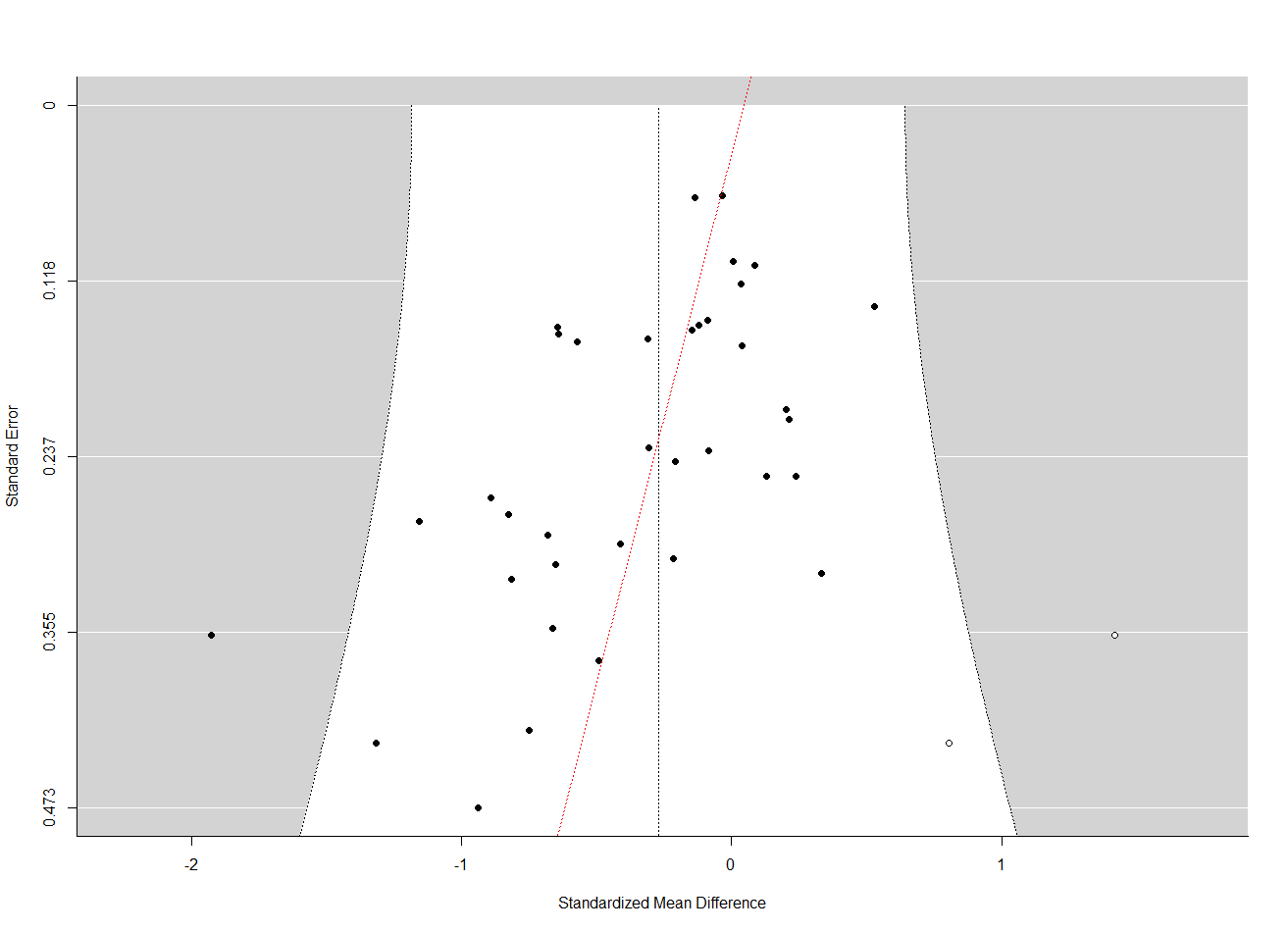

Supplement: Multimedia Appendix 7 [file mental_v8i11e32199_app7.docx]
